# High-performance graphene/aluminum composites: progress and challenges from an industrial perspective

**DOI:** 10.3762/bjnano.17.86

**Published:** 2026-09-14

**Authors:** Shuai GuoLiang, Pan LinXia, Ma HaiLin

**Affiliations:** 1 Postdoctoral Research Station, Xinjiang Joinworld Co., Ltd., No. 18, Kashi East Road, High-tech District, Urumqi, Xinjiang,China; 2 Xinjiang Chemical Engineering Design Institute, State Power Investment Co. Ltd., No. 559, Kashi East Road, High-tech District, Urumqi, Xinjiang,China; 3 Stevens Institute of Technology, Hoboken, NJ 07030, USA

**Keywords:** graphene, graphene/aluminum composite, industrial manufacture

## Abstract

Graphene/aluminum (Gr/Al) composites are regarded as a promising class of lightweight, high-performance materials for aerospace, automotive, and electronics applications. Graphene’s exceptional mechanical, thermal, and electrical properties are integrated with aluminum’s favorable density and formability. From an industrial perspective, different methods for producing graphene are analyzed, and graphene/aluminum composites are discussed. The industrialization bottlenecks and path strategies for high-performance Gr/Al composites are analyzed. The major bottlenecks are uniform dispersion of graphene in Al matrix, suppression of the harmful Al_4_C_3_ interfacial phase, and cost control for large-scale production. The former two problems are addressed by the development of low-cost graphene surface modification technology. The latter problem can be solved by combining different composite technologies, such as spark plasma sintering and casting, which enables excellent microstructure controllability and efficiency. This dual-path framework is provided as a practical reference for the commercialization of Gr/Al composites.

## Review

### 1 Introduction

As an emerging class of lightweight, high-performance materials, graphene/aluminum (Gr/Al) composites have attracted considerable scientific and engineering interest in recent years. Exceptional promise for advanced applications in aerospace engineering, automotive manufacturing, and electronic packaging systems is exhibited by these novel composites [1–2]. Scientific inquiry into these materials began after the seminal findings regarding mechanical graphene exfoliation methodologies in 2004 [3]. The inaugural theoretical investigation concerning graphene/aluminum composites was documented in 2008 and was succeeded by the first experimental validation employing powder metallurgy techniques in 2011 [4–5]. Graphene’s extraordinary intrinsic properties, namely, its high theoretical tensile strength (approximately 125 GPa), exceptional thermal conductivity (circa 5000 W·m^−1^·K^−1^), superior electrical conductivity (approximately 100·10^6^ S·m^−1^), and substantial specific surface area (approximately 2630 m^2^·g^−1^), are integrated with aluminum’s favorable density characteristics and formability attributes. Thereby, an optimal material solution for structural–functional integration requirements is provided [6–14]. Within the global paradigm of energy transition and sustainable development, raised requirements for lightweight structural components, advanced thermal management solutions, and efficient power transmission/conversion systems in transportation sectors have directly accelerated the rapid advancement of graphene/aluminum-based composite technologies. These composites are established as a research focus in today’s materials science [15–16].

Despite their considerable potential, three fundamental technical barriers are encountered in the practical implementation of these composites; these are interfacial bonding and control mechanisms, homogeneous dispersion methods, and the realization of synergistic multiproperty performance enhancement [17–20]. During composite fabrication processes, brittle aluminum carbide (Al_4_C_3_) phases are frequently generated by interfacial reactions between graphene constituents and the aluminum matrix. The functional role of this interfacial phase remains contentious within the scientific literature: Al_4_C_3_ is proposed by certain investigations to enhance composite strength through robust interfacial bonding, whereas its propensity to initiate brittle fracture at material interfaces is indicated by alternative studies [21–24]. Then, owing to substantial van der Waals interactions between adjacent graphene layers at the nanoscale, there are significant challenges in achieving homogeneous dispersion within the aluminum matrix. This tendency toward agglomeration readily establishes crack nucleation sites while electron transport and thermal conduction pathways are concurrently impeded [25–27]. Furthermore, performance trade-offs between strength and toughness, strength and electrical conductivity, or strength and thermal conductivity parameters are typically exhibited by conventional composite systems, wherein strength enhancement invariably compromises ductility, electrical conductivity, or thermal transport capabilities respectively [20,22].

A comprehensive “preparation–interface–performance–application” framework is adopted in this review to systematically evaluate recent advances in graphene/aluminum-based composites over the preceding decade. The analysis encompasses preparation methodologies, interfacial engineering strategies, performance characterization methods, and application prospects. Additionally, contemporary academic understanding of interfacial interaction mechanisms and interfacial modification approaches is emphasized, alongside innovative solutions progressively addressing these persistent challenges. Theoretical foundations for future research trajectories and prospective industrial applications are established as the objective.

### 2 Graphene preparation and dispersion

The performance of graphene/aluminum composites is fundamentally tied to how they are made. Three key aspects are directly impacted by different processing routes, that is, how well graphene is bonded with the aluminum matrix, whether the graphene sheets are spread evenly throughout the material, and what final properties are obtained [1,6,17]. From research and industry experiences, the fabrication can be broken into three stages, namely, graphene is synthesized, it is properly dispersed, and finally the composite is formed [18–20]. The real breakthrough happens when these steps are optimized together rather than separately.

**Figure 1:**
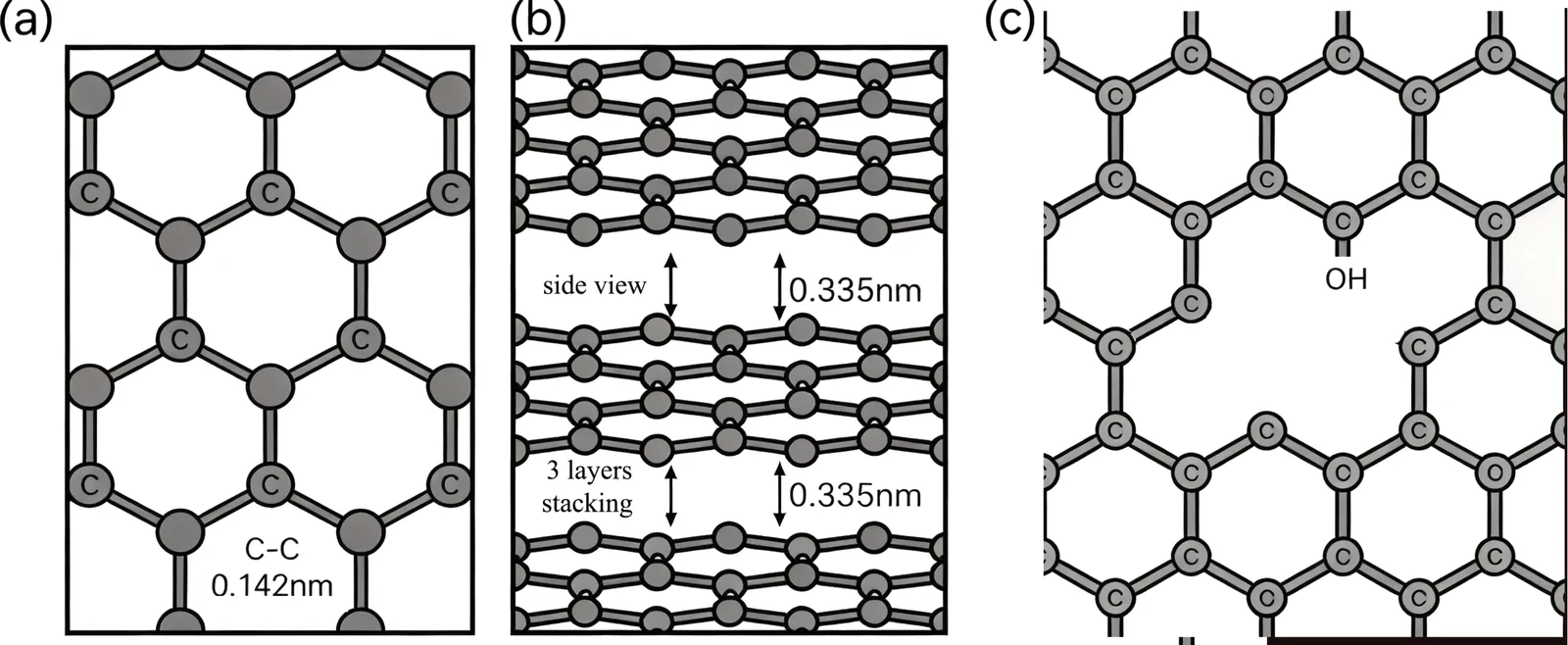
A schematic showing the crystal structure of graphene materials: (a) pristine graphene, (b) three-layer graphene, and (c) reduced graphene oxide.

Graphene is the two-dimensional crystalline allotrope of carbon, with the atoms arranged in a honeycomb lattice configuration [9]. Graphene materials are typically classified based on their layer count, structural integrity, and the presence of functional groups or defects [9,27–34]. Single-layer graphene consists of a one-atom-thick carbon lattice, while few-layer graphene refers to stacks of two to ten layers [9,28]. Structural quality is another key criterion; it is distinguished between pristine, defect-free graphene and materials containing vacancies, grain boundaries, or chemical modifications [29–31]. Additionally, graphene derivatives such as graphene oxide (GO) and reduced graphene oxide (rGO) are defined by their oxygen-containing functional groups and subsequent reduction processes [32–34]. The different crystal structures of the three different graphene materials are shown in Figure 1. Precise communication regarding material properties and performance across research and industrial applications is enabled by this systematic classification. These structural attributes and corresponding application domains are comprehensively summarized in Table 1 [9,27–34].

**Table 1:** Classification and characteristics of graphene [9,27–34].

Classification	Type	Structural features	Typical applications

layer number	monolayer graphene	single-atom-thick honeycomb lattice (thickness ≈ 0.34 nm)	high-frequency electronics, sensors
	bilayer graphene	AB/AA stacking with interlayer coupling effects	field-effect transistors
	few-layer graphene (3–10 layers)	enhanced dielectric screening, tunable carrier density	energy storage electrodes, composites
	multilayer graphene (>10 layers)	graphite-like properties, reduced conductivity/thermal conductivity	thermal films, structural reinforcements, power transmission

functionalization	graphene oxide (GO)	oxygen-containing groups, hydrophilic, reduced conductivity	biosensor, water purification membranes
	fluorinated/hydrogenated graphene	chemical surface modification for magnetism/hydrophobicity	spintronics, anticorrosion coatings

morphology	film	CVD-grown, superior transparency (>97%) and conductivity	transparent electrodes, flexible displays
	powder	mechanical exfoliation or redox synthesis, high specific surface area (2630 m^2^·g^−1^)	battery additives, supercapacitors
	nanoribbons/quantum dots	edge-dominated quantum confinement effects	quantum computing, optoelectronics

Graphene can be synthesized through a variety of techniques, which are mainly segregated into two conceptual categories, namely, “bottom-up” approaches, wherein atomic precursors are coalesced to form monolayer structures, and “top-down” techniques, wherein bulk graphite is progressively exfoliated into thinner configurations [3,35–38]. Chemical vapor deposition (CVD) is widely employed to produce high-quality, large-area graphene films on metal substrates such as copper or nickel [35]. Alternatively, single-layer graphene flakes are isolated from bulk graphite by mechanical exfoliation, also known as the “Scotch tape” method [9]. Additional fabrication routes include liquid-phase exfoliation, where graphite layers are delaminated with the assistance of solvents or surfactants, as well as epitaxial growth on silicon carbide (SiC) substrates [3,36–38]. A versatile toolkit for tailoring the structural and functional characteristics of graphene according to specific application requirements is collectively provided by these methods. Significant variations in defect density, structural characteristics (including charge carrier mobility), and production efficiency/cost parameters are exhibited by graphene produced via these distinct approaches, as systematically documented in Table 2 [3,9,35–38].

**Table 2:** Performance-cost analysis of main industrial graphene synthesis methods [3,9,35–38].

Method	Key process	Product characteristics	Cost	Industrial maturity

liquid-phase exfoliation (LPE)	water-jet shear peeling	highest quality (defects <0.1%), size <100 μm, layers>10 in generally by LPE	low by LPE	mass production by LPE
redox method	Hummers’ oxidation + chemical reduction	oxygen residues (C/O≈8:1), conductivity 10^2^ S/m	low	mass production
CVD	CH_4_ decomposition on Cu/Ni (1000°C)	large-area (square meter scale), mobility >4000 cm^2^·V^−1^·s^−1^	medium (film)	pilot-scale
SiC epitaxy	SiC annealing (>1400°C)	transfer-free but nonuniform thickness	high	research and development

Several industrial-scale methods exist for graphene production, including the oxidation–reduction of graphite (redox method) and liquid-phase exfoliation (LPE), each with distinct advantages and limitations. In the CVD technique, metal substrates are exposed within a reaction chamber to hydrocarbon gases and hydrogen at elevated temperatures. Carbon atoms are decomposed from the gas precursors and are crystallized into a graphene film on the metal surface [35]. A critical subsequent step involves transferring the graphene material from the growth substrate, and challenges in production efficiency occur. Therefore, the redox method of graphite was developed, in which graphite is chemically oxidized using strong acids and oxidants to produce GO. GO is hydrophilic, and its dispersion and processing in aqueous solutions are facilitated. The graphene structure is subsequently restored by reduction (via chemical agents or thermal treatment), yielding reduced graphene oxide. However, residual defects and oxygen functionalities are typically contained in rGO, and its mechanical and electrical properties are compromised compared to CVD graphene [32–34,39]. The initial mechanical exfoliation is the original Scotch tape method, in which layers are mechanically cleaved from bulk graphite [9]. While instrumental for fundamental research, it suffers from extremely low yield and poor scalability, which render it unsuitable for industrial manufacturing. LPE was subsequently developed as a mechanical exfoliation method. Graphite powder is dispersed in specific solvents or surfactant solutions. High-energy input, such as ultrasonication or shear mixing, is applied to exfoliate graphite into graphene flakes. While potentially scalable, precise control over flake size and thickness (number of layers) is difficult with LPE, and post-processing is often required to concentrate the material [40].

Redox method and LPE are also the two main industry methods. rGO is the current mainstream industrial product for graphene as its preparation process has matured to relatively low requirements regarding equipment, leading to low cost. However, strong acids and strong oxidants are required for its preparation, resulting in a large number of defects in the obtained graphene and environmental pollution. In addition, its precursor graphene oxide possesses excellent hydrophilic dispersibility coupled with mature process technology and high batch consistency, which enable good manufacturability. Graphene obtained via LPE has an intact crystal lattice and few defects, with a yield second only to that of rGO. However, size and thickness distributions of the exfoliated sheets are difficult to be controlled, and high requirements on post-treatment are imposed by large-scale production, making it more suitable for functional applications with extremely high quality requirements. Key challenges across these methods include balancing production cost, achieving high material quality (e.g., low defect density and large domain size), and ensuring consistent layer control, as shown in Table 3 [38–44].

**Table 3:** Comparison of redox reduction and liquid-phase exfoliation in terms of efficiency, structural characteristics, and applicable scenarios [38–44].

Parameter	Redox method	Liquid-phase exfoliation

production rate	0.27–0.42 tons per day for the single-line production capacity	up to 0.4 tons per day for the single-line production capacity
defect rate	relatively high (residual oxygen-containing functional groups)	extremely low (preservation of complete sp^2^ structure)
structural characteristics	surface with wrinkles and topological defects	multilayers, thickness control requires centralized post-processing.
applicable scenarios	low-cost batch production	high-performance composite materials (such as power transmission and heat dissipation)
cost	88–147 US$/kg for few-layer graphene	~250 US$/kg for few-layer graphene

Recent breakthroughs in graphene dispersion techniques range from ultrasound applications to cryogenic techniques and self-assembly [44–46]. GO clusters were dispersed by Wang et al. [45] using high-frequency sound waves. When just 0.3 wt % of the obtained GO was mixed into aluminum, a 62% jump in strength was shown. Han et al. [46] used a cold environment of 193 K in which graphene sheets were stopped from folding and stacking. With a 10:1 ball-to-powder ratio and 300 rpm rotation speed, graphene stacks only five atoms thick were obtained while the cold welding problem was avoided. Yang et al. [47] made use of electrostatic forces, and graphene was neatly arranged onto aluminum powder. After hot-pressing, a layered structure was developed by the composite, almost like mother-of-pearl. With only little added graphene (0.083 vol %), the material gained strength without sacrificing its stretchiness, which is still over 20% elongation after fracture. From the perspective of industry and production, the selection of graphene for graphene/Al composites requires a comprehensive balance among cost, performance, dispersion process, and interfacial compatibility with the matrix. Reduced graphene oxide and graphene from LPE are the most suitable materials for general application. Reduced graphene oxide is currently regarded as one of the most promising reinforcing materials for mass production. However, its laminar structure contains abundant defects and oxygen-containing functional groups; while these features are beneficial for chemical modification and interfacial bonding, electrical and thermal conductivity are significantly impaired, which limits applications of rGO.

Graphene obtained via LPE has an intact crystal lattice and excellent intrinsic electrical and thermal conductivity, but it suffers from a wide layer-number distribution. Also, it is more expensive than rGO, making it more suitable for application scenarios with high functional requirements, such as conductive and heat-exchange composites. In terms of layer number selection, the best theoretical strengthening effect can be achieved with single-layer or few-layer graphene, which are expensive, highly prone to agglomeration in matrix composites, and more likely to react with aluminum at high temperatures to form Al_4_C_3_, by which interfacial strength is deteriorated. Our practical research shows that a good balance between strengthening efficiency and dispersion uniformity can be achieved by the selection of multilayer graphene with a thickness of 2–20 nm. Intense interfacial reactions are avoided, and strength and electrical/thermal conductivity are improved via grain boundary pinning and load-bearing effects.

In addition, to improve the bonding between graphene and aluminum melt and to control interfacial products, rGO can be modified with copper, nickel, or titanium. Also, graft modification can be conducted using the oxygen-containing groups of rGO; thus, agglomeration is reduced and the formation of harmful carbides is inhibited.

Therefore, in current industrial routes, cost-controllable rGO with modification treatment is preferred for mechanically reinforced composites. For products requiring both high electrical conductivity and high thermal conductivity, few-layer or multilayer graphene prepared by mechanical methods may be used, and uniform distribution is achieved via process optimization after surface alloying.

### 3 Gr/Al composite fabrication methods

#### 3.1 Conventional fabrication methods

Several techniques are involved in the fabrication of graphene/aluminum composites. Each technique brings distinct benefits for structure control, production scaling, and performance boosting. Key problems are tackled by these approaches, including uniform graphene distribution and strong interfacial bonding, through smart engineering solutions. Currently, leading industrial techniques are broadly categorized into solid-state and liquid-phase categories [38,48–52].

Solid-phase methods dominate graphene/aluminum composite manufacturing. Mechanical energy is used to mix graphene into the aluminum matrix. A big advantage is found in the lower working temperatures and controllable reactions at the interface [53]. These features make those methods well suited for parts that need fine grains and high density. Spark plasma sintering (SPS) and accumulative roll bonding (ARB) are the core techniques here. Sufficient flexibility is provided for handling different starting materials and performance targets [24,48–52].

SPS works by first anchoring graphene nanoplatelets (GNPs) onto aluminum powder using high-energy ball milling. Pulsed electric currents are then applied during sintering to create heat and pressure. This fast process densifies the material at surprisingly low temperatures (well below aluminum’s melting point) in just 5–10 min [24,49]. Because of the short processing time, grains cannot grow large. Ultrafine grains smaller than 1 µm average result [24]. Nearly full density (>99.2%) with very little remaining porosity (0.56–0.75%) can be achieved by SPS at 500 °C using proper milling time (10 h) and pressure (20–50 MPa) [53]. However, SPS is not perfect. Its equipment is pricey and part size is limited. Special power supplies and vacuum setups are needed for SPS equipment, costing three to five times more than standard hot presses. Part size is also constrained by the mold design and current flow uniformity. Currently, SPS parts (such as ceramic) up to 100–300 mm can be made by industry, but their shape (aspect ratio) is limited, usually to 2:1 or less [54].

ARB takes a different route. Layers of aluminum foil are stacked with graphene oxide sprayed in between, then rolled together. Typically, a GO/water mixture is sprayed onto the aluminum foil, and 5–8 layers (each 0.1–0.3 mm thick) are stacked. After cold rolling, the thickness is reduced by 50–70% [55–58]. and a layered structure has formed. The intense shear during rolling (reaching 5–8%) helps align the GO along the aluminum grain boundaries. The yield strength of Al6061 is boosted to 320 MPa (a 65% increase) by adding just 0.2 wt % GO this way. Reasonable stretch (12% elongation) is kept, and fracture toughness is improved to 28 MPa·m^1/2^. Grain refinement is also achieved by ARB (the grain boundary area is tripled), and GO acts as a barrier. Together, the corrosion rate in salt water (3.5% NaCl) is decreased down to 0.0859 mm per year, an 89% drop compared to the matrix alloy [55]. However, ARB has its own challenges, including interfacial waviness and edge cracking. Bonding strength can vary a lot across the interface if the rolling temperature is not steady [56–58]. Precise temperature control systems, like infrared feedback loops, are often needed for this reason [59]. Also, microcracks tend to form at the edges if the rolling layers are too much in one pass (over 75% reduction). Laser trimming is usually required to fix this and decrease material losses.

Liquid-phase processing provides essential pathways for fabricating Gr/Al composites. Graphene is dispersed directly within molten metal by these methods, enabling large-scale production of complex-shapes. Energy use is cut by 30–40% compared to solid-state approaches, and near-net-shape forming is supported. Two main techniques dominate, namely, semi-solid electromagnetic stirring and conventional stir casting. Each fits different production requirements [17,19,26,60–64]. Semi-solid electromagnetic stirring combines ball milling with magnetic fields. Alternating magnetic fields, usually 30–60 Hz, generate Lorentz forces, and eddy currents create shear rates of 50–100 s^−1^ [62–64]. Graphene clusters are broken up uniformly by this mechanism. Andilab et al. [62] successfully prepared GNP/A319 aluminum alloy composites using ultrasonic-assisted semi-solid casting. The ultimate tensile strength, yield strength, and elongation were enhanced by 10%, 11%, and 32%, respectively, by the addition of 0.05 wt % GNPs. Uniform distribution of GNPs within the aluminum matrix with favorable interfacial bonding was revealed by SEM observations, with no significant agglomeration or Al_4_C_3_ phase formation. The strengthening mechanism was primarily attributed to the Hall–Petch effect, followed by coefficient of thermal expansion mismatch and load transfer [62]. Furthermore, high scalability potential is demonstrated by this process. Continuous production rates exceeding 200 kg·h^−1^ are enabled by multistation electromagnetic stirring systems. The critical challenges of this method involve porosity control and parameter sensitivity management. Gases are easily trapped by melt turbulence, causing 1.8–3.5% porosity without vacuum (≤10^−2^ Pa) or hot isostatic pressing (HIP) treatment, far above standard casting’s 0.5% [62–64]. Besides, graphene distribution is disrupted by power fluctuations without precise PID control.

The stir casting process incorporates rapid mechanical mixing of graphene into molten aluminum (680–720 °C) using rotating impellers, followed by conventional casting operations. Equipment investment approximately one-fifth that of electromagnetic stirring systems is required by this methodology, making it suitable for small-to-medium batch production scenarios [27,60–61]. However, poor graphene wettability in molten aluminum environments promotes agglomeration under limited mechanical shear conditions. Agglomerate dimensions frequently reach 20–50 µm at 1 wt % GNP incorporation levels, reducing composite elongation to 40% of the matrix alloy value [65]. Additionally, Al_4_C_3_ formation (content up to 2.1 vol %) is promoted by elevated processing temperatures (>700 °C) [38].

Recommended processing methods for graphene/aluminum composites under different application scenarios and requirements are shown in Table 4 as a reference for industrial manufacturing. While vital for industrial production, liquid-phase methods have clear limits regarding the product properties. Future work should combine SPS with electromagnetic stirring–extrusion to tackle porosity and boost output efficiency simultaneously. It is worth noting that powder metallurgy sintering equipment (such as SPS and HIP) has enabled great technical progress in the past 2–3 years driven by the strong demand for large-format, high-throughput production of dense solid-state batteries electrolytes and electrodes; all the aforementioned sintering technologies are now rapidly evolving toward larger processing dimensions, ultrafast sintering capability, and continuous production mode. The iteration of these equipment has greatly promoted the development of graphene/aluminum alloys.

**Table 4:** The recommended processing methods for graphene/aluminum composites under different application scenarios and requirement.

Application requirement	Recommended process	Advantages	Typical application scenario	Maximum producible component size/weight

ultrahigh strength	SPS (0.3–1.2 wt % GNPs)	grain refinement (0.8 μm), density >99.2%	aero-engine components	400 × 400 × 600 mm, ~200 kg
complex thin-walled structures	semi-solid electromagnetic stirring	equiaxed crystal structure (shape factor 0.84)	new energy vehicle battery casings	10 g to 100 kg
corrosion resistance priority	ARB (0.1–0.5 wt % GO)	corrosion rate reduced by 89%	marine engineering structures	sheets of less than 100mm in width generally
low-cost mass production	stir casting	unit cost below US$25	electronic packaging substrates	diameters of 70–520 mm in generally, same as in direct chill casting

Among these traditional processing methods, stir casting has a higher level of industrial maturity and is most suitable for mass production. SPS is applicable for high-end, small-batch manufacturing, where superior properties justify the higher processing cost. Remarkable advantages in cost are found in stir casting with about US$1,500 per ton (Table 5). With relatively low equipment investment and mature process routes, large-size production is easily realized, and excellent compatibility with existing smelting and casting systems of the aluminum industry is achieved. The dispersion of graphene in the melt can be improved to a certain extent by optimizing process parameters such as ball-milled preforms and electromagnetic stirring, and synergistic improvement of strength and plasticity is achieved. This makes stir casting a feasible industrial route for mass, low-cost preparation of Gr/Al composites with moderate performance requirements. Its shortcomings are also obvious: Intense interfacial reactions occur without interface modification of graphene, leading to limited improvement in strength, electrical conductivity, and thermal conductivity of the composites. Consistency control of wire products along the length direction is acceptable, but that of large-section cast products is poor.

**Table 5:** Cost breakdown of Gr/Al composites prepared by different routes (excluding the cost of aluminum; benchmarks: 2000 t/a annual production capacity, 0.1–0.5 wt % few-layer graphene loading, unit: US$ per ton, all relevant prices are based on the Chinese market).

Cost item	Stir casting route	SPS route

raw material cost (few-layer graphene)	~900	~900
utility (fuel and power) cost	~88	~60
plant depreciation cost	~100	~300
equipment depreciation cost	~180	~4800
labor cost	~240	~3600

total cost	~1508	~9660

Prominent advantages regarding process stability and microstructure control are found in the SPS route. The formation of the harmful interfacial phase Al_4_C_3_ can be effectively inhibited via the parameters of rapid heating/cooling and pressure-assisted sintering. Although the cost of SPS equipment is relatively high, high process accuracy, strong repeatability, and precise regulation of graphene dispersion and interfacial bonding can be achieved to obtain products with excellent performance. Takt production rather than continuous production is adopted by SPS, so the processing fee is usually above US$9,000 per ton (Table 5) with lower production efficiency, which increases the investment of human resources and equipment. It is especially suitable for scientific research exploration and the production of high-performance, high value-added products, such as high-strength structural components and thermal dissipation materials for AI servers. Besides, it can be seen that the cost of the graphene raw material is not the major expense.

#### 3.2 Advanced novel fabrication strategies

Significant technological advancements in graphene/aluminum composite manufacturing have been observed in recent years. Two methods stand out, namely, additive manufacturing, especially laser powder bed fusion (LPBF), and deformation-driven metallurgical techniques [20,66–70]. LPBF works by melting layers of graphene/aluminum powder with a powerful laser (200–400 W). The powder is melted quickly, and graphene is trapped inside the aluminum as it cools down fast. Finer grains in the metal form due the graphene addition, and the metal is made much stronger by effective load sharing at the interface. To start, the powder mix is made uniform using ball milling or chemical coating, typically adding between 0.1 and 3.0 wt % graphene. The right process parameters are crucial, that is, layer thickness around 30 µm, laser speed of 800–1200 mm·s^−1^, and laser path overlap of 20–40%. Careful control is applied to stop graphene from breaking down too much [20,66–70]. Exceptional mechanical enhancement, multifunctional properties, and controlled anisotropy are demonstrated by materials fabricated through this methodology. Tensile strength exhibits a more than 40% improvement relative to pure aluminum, attributable to synergistic dislocation pinning and Orowan strengthening mechanisms [66–68]. While additive manufacturing incurs higher initial capital investment, material waste is substantially reduced to approximately 5% (compared to 20% in conventional casting processes), and total lifecycle costs are consequently diminished [20,66–68].

**Figure 2:**
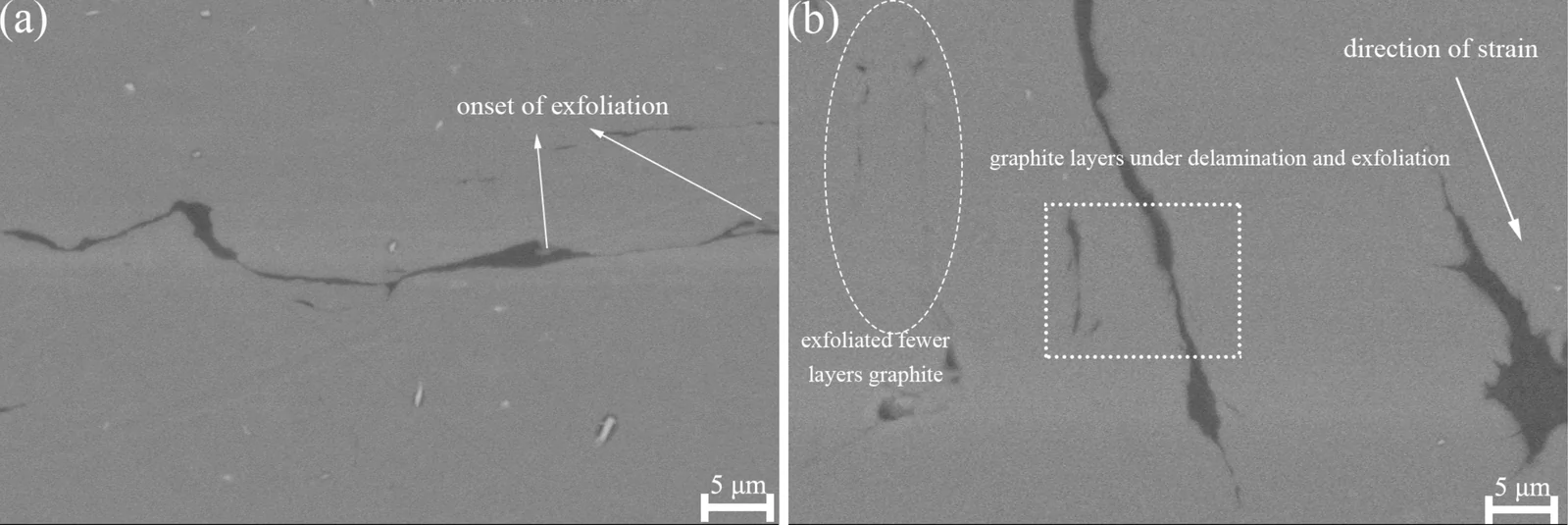
Graphene from graphite in metal matrix composites via severe plastic deformation.

Deformation-driven metallurgical bonding utilizes intense shear stresses generated during severe plastic deformation (SPD) to exfoliate graphene from graphite precursors in situ. Deformation-induced thermal energy is simultaneously harnessed to establish metallurgical bonding between graphene and the metallic substrate [69,71–72]. In situ graphene exfoliation coupled with dynamic recrystallization of the aluminum matrix was achieved by Huang et al. [73] through high-strain deformation (ε > 5) via friction stir processing (FSP) and associated frictional heating, within a one-minute processing window. A tensile strength of 468 MPa (293.3% enhancement) and exceptional ductility of 19.9% were imparted to the 1.5 wt % GNPs/Al composite [73]. Multipass SPD extrusion (3–7 passes) conducted at 500 °C was employed by Wu et al. [74] to achieve in situ exfoliation of graphite flakes. Shear deformation was exploited to induce interlayer slippage and dissociation of graphite structures, yielding graphene, while dynamic recrystallization was promoted to generate ultrafinely grained microstructure [74]. Similar results were also observed after the processing of our 1% graphite/aluminum composite after severe plastic deformation, as shown in Figure 2.

Graphene/aluminum composite manufacturing technologies are now largely in the early stages of industrialization (Table 6). Low cost and good compatibility with existing infrastructure are offered by liquid-phase routes (stir casting or semi-solid electromagnetic stirring), but dispersion challenges limit their technology readiness levels (TRL), leaving a gap to reliable application. Superior interfacial control is provided by solid-phase routes (SPS or SPD), with SPS reaching the highest TRL of 4–5 and already being suitable for high-value products at a high price. Great promise is held by LPBF, but it remains the least mature technology. The key to industrialization lies in achieving uniform graphene dispersion by interface modification, which is discussed in Section 4, and establishing robust process windows, thereby enabling the transition from laboratory specimens to industrial products.

**Table 6:** Comparison of manufacturing technologies for graphene/aluminum composites.

Manufacturing technology	Technology readiness level	Applicable industrial product types

spark plasma sintering	4–5	high-value precision components, such as aerospace sensors, thermal management devices, and electronic packaging shells
semi-solid electromagnetic stirring	3–4	automotive brake discs, heat sinks, and cost-sensitive castings with relatively simple geometries
stir casting	4–5	low-to-medium-end heat-exchange parts and structural supports, where moderate uniformity can be tolerated
laser powder bed fusion	3–4	customized functional parts with extreme geometric complexity, such as lattice-structured heat sinks and lightweight aerospace brackets
severe plastic deformation (including ARB and FSP)	2–3	high-strength thin sheets, surface strengthening of structural components subjected to harsh local service conditions, such as piston crowns and die surfaces

### 4 Interface engineering and properties change mechanisms

#### 4.1 Interface engineering strategies

**Figure 3:**
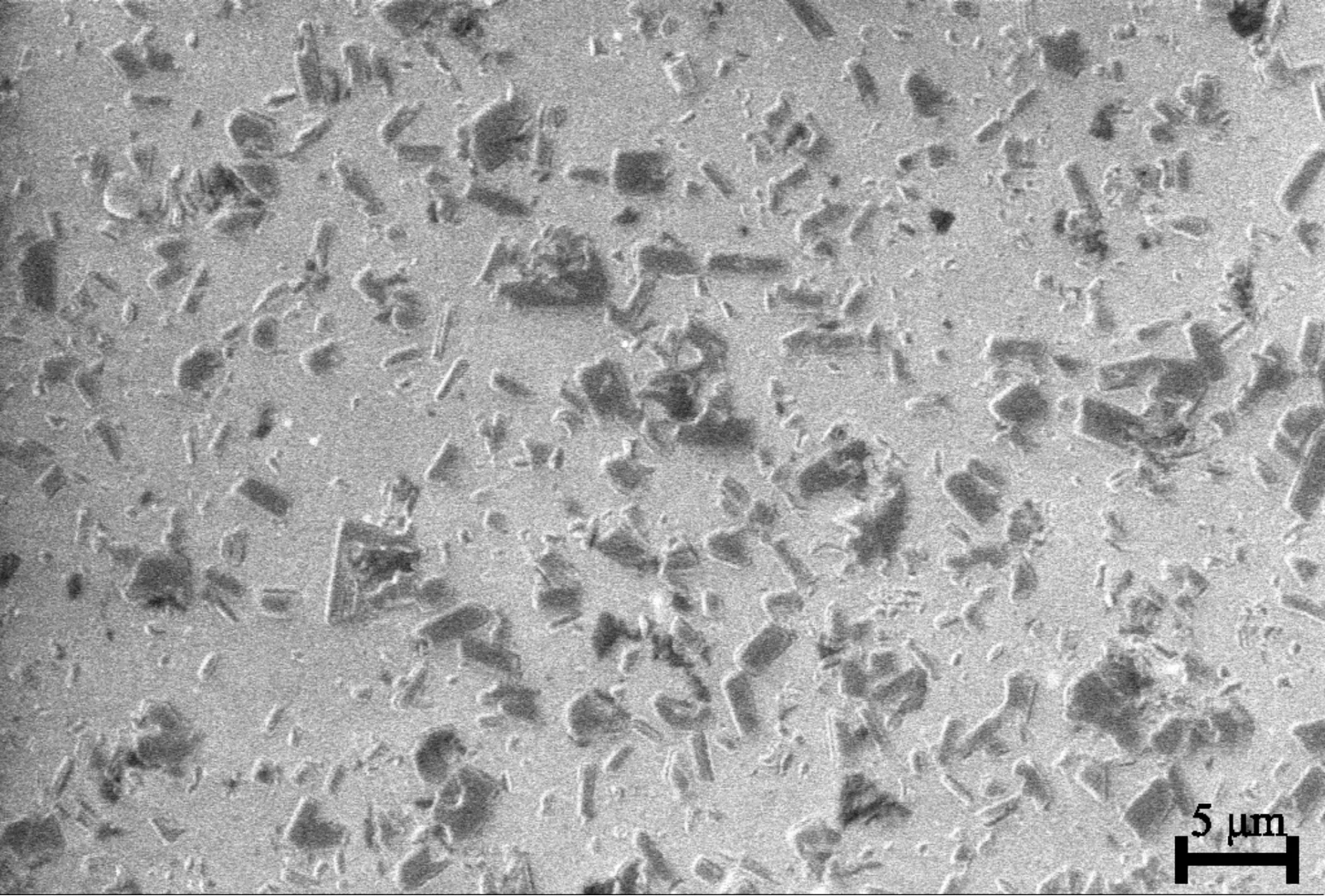
SEM image of Al_4_C_3_ in a 1% Gr/Al composites sintered at a temperature of 650 °C from our work.

The interface between graphene and the aluminum matrix is a defining microstructure that critically influences the overall performance of graphene/aluminum composites. Significant progress in both experimental characterization and computational modeling has been made, and this has deepened the understanding of these interfacial regions and their fundamental behaviors [20,38,45,75–80]. One of the key features at the graphene/aluminum boundary is the formation of the Al_4_C_3_ phase. The typical microstructure of Al_4_C_3_ is shown in Figure 3, the morphology is short rod-like/fragmented, protruding after polishing due to high hydrolysis susceptibility. Based on molecular dynamics simulations [75–77], the formation kinetics of Al_4_C_3_ at graphene/aluminum interfaces is found to follow a diffusion-reaction mechanism. Above 450 °C, aluminum atoms are observed to diffuse through graphene edge defects to form tetrahedrally arranged Al–C bonds, and the reaction kinetics is dominated by three factors. Within the temperature range of 550–630 °C, the rate constant is found to adhere to an Arrhenius relationship with an activation energy barrier of 156 kJ·mol^−1^ for atomic migration and bond reorganization. The crystallographic orientation was shown to significantly modulate reactivity, the prismatic facets exhibit 40% higher activity than the basal (0001) planes due to abundant step edges and dangling bonds, whereas the dense atomic arrangement of the planes imposes greater kinetic resistance. Defect engineering was found to play a catalytic role; single vacancies reduce the reaction energy barrier by 0.8 eV (~77.2 kJ·mol^−1^) by releasing local strain and exposing highly reactive carbon atoms [78]. These atomic-scale insights enable precise control of interfacial Al_4_C_3_ growth through synergistic optimization of temperature, defect density, and graphene orientation.

It is crucial to note that excessive Al_4_C_3_ formation is detrimental and leads to interfacial brittleness and heightened susceptibility to degradation by moisture [20,38]. However, the precise functional role of Al_4_C_3_ remains a topic of active debate. To probe the atomic-scale formation dynamics of Al_4_C_3_, a neural evolutionary potential (NEP) model was developed by researchers [81]. Simulations based on this model suggest that controlled formation of Al_4_C_3_ at the interface can be beneficial. It appears to strengthen interfacial bonding by creating a network of robust covalent bonds. Molecular dynamics simulations leveraging the NEP potential revealed that composites with such Al_4_C_3_-modified interfaces exhibit impressive mechanical properties. Under tensile loading parallel to the interface, ultimate strengths greater than 2 GPa were achieved [81]. This represents a substantial increase compared to pure aluminum. Analysis of shear deformation further showed that the covalent interfacial bonds significantly raise the energy barrier for atomic slip approximately threefold. Consequently, these composites are observed to combine exceptional strength with retained ductility levels of 15–25%, and this balance is often difficult to achieve.

The key factor determining the influence of Al_4_C_3_ is its quantity at the interface. When the processing temperature is controlled within the range of 450–630 °C, and the holding time is kept short, very little Al_4_C_3_ forms in the form of discontinuous nanoscale particles at the interface. In this case, the beneficial effects are dominant. It has been shown by studies that the interfacial reaction rate between graphene and aluminum is very slow below 600 °C, and only small quantities of fine Al_4_C_3_ are yielded. These fine Al_4_C_3_ nanoparticles can serve as chemical anchoring points at the interface, and the originally weak physical bonding is transformed into locally strong chemical bonding. Thereby, the load transfer efficiency is enhanced. Therefore, under low-temperature solid-state processing or short-duration rapid sintering conditions, the thermal input is insufficient to drive excessive Al_4_C_3_ reaction. The interfacial reaction products are limited, and the beneficial effects dominate the material properties. When the processing temperature exceeds the melting point of aluminum (660 °C) or prolonged high-temperature holding is applied, the two-dimensional structure of graphene suffers severe damage. The reaction rate between graphene and aluminum is increased significantly, and this leads to rapid and massive formation of Al_4_C_3_. At this point, the detrimental effects sharply take the dominant role. Table 7 shows the comparison of different graphene/aluminum composite processing methods from the perspective of Al_4_C_3_ formation.

**Table 7:** Interfacial reactions in main synthesis methods of graphene/aluminum composites.

Method	Temperature range (°C)	Graphene content (vol %)	Interfacial reactions	Typical performance enhancement

ball milling + hot pressing	400–600	0.5–5.0	prone to Al_4_C_3_ formation	47% increase in hardness [44]
melt ultrasonic dispersion	660–800	0.1–2.0	high amounts of Al_4_C_3_, requires barrier coating	75% increase in thermal conductivity [39]
multipass friction stir processing	400–500	1.0–3.0	no Al_4_C_3_ formation	threefold increase in yield strength [38]
vacuum-assisted infiltration	700–900	3.0–10.0	high amounts of Al_4_C_3_, requires preform	230% increase in flexural strength [20]

To decrease the harmful interfacial reactions, multiple interfacial modifications have been suggested by researchers. In direct Al/graphene reactive interfaces, strategic modulation of sintering temperature and dwell time facilitates controlled formation of nanoscale Al_4_C_3_ at the Al/graphene interface, and robust covalent bonding networks are established. It has been established by research that the (001)Al∥(0001)Al_4_C_3_ orientation relationship yields optimal mechanical properties [21,81–82]. Intermediate-phase modified interfaces incorporate Al_2_O_3_ or Al_4_SiC_4_ as transition layers, and this enables controlled load transfer while interfacial bonding strength is enhanced and deleterious interfacial reactions are suppressed [82]. Besides, coating metals on graphene layers are considered an effective method to solve the poor interface bonding [82]. Ni is the best coating metal, enhancing interfacial strength by 145.34% due to the highest value of pullout force. Additionally, three-dimensional topological interfaces have been developed. Inspired by origami principles, graphene origami (GOri) structures were designed by researchers [83]. Uniform stress distribution along crease edges is ensured through this configuration by specific unfolding behavior. The distinctive folded topology of GOri has demonstrated a 37% enhancement in indentation load with a 138% increase in indentation depth, and stress concentration phenomena inherent to conventional two-dimensional graphene architectures are effectively mitigated.

#### 4.2 Property-change mechanisms

It has been confirmed by researches that graphene addition significantly improves the strength and hardness of aluminum matrix composites [17,20,22,38]. Innovative interface design further allows for simultaneous improvement of both strength and ductility. The strengthening mechanisms in graphene/aluminum composites include not only those seen in common aluminum alloys, such as fine-grain strengthening, dislocation strengthening, particle strengthening, and solid solution strengthening, but also effects unique to graphene itself. These specific mechanisms from graphene involve load transfer strengthening, grain refinement induced by graphene, Orowan strengthening mediated by graphene, and strain hardening by graphene [17,20,22,38].

Shear lag theory says that when the interfacial bonding strength reaches a critical level, external loads are effectively transferred to graphene reinforcements through interfacial shear stress. This explains the load transfer [45,48,51]. These findings are supported by experimental studies on GNP/Al composites made by ball milling and semi-solid stirring. Adding GNPs increased composite hardness by ~40% and tensile strength by ~35%. This improvement comes from combined effects of GNP load transfer strengthening and grain refinement [48,51]. Graphene is also found to act as a nucleation site for aluminum crystallization while grain boundary movement is blocked during processing [38,84]. This causes significant grain refinement. In AA-3003/GNPs and AA-5154/GNPs composites, fast solidification, and non-equilibrium nucleation shrank grains to sub-micrometer sizes [85]. Besides, dislocation motion is blocked by graphene, and dislocations accumulate at the interfaces. Higher stress is needed for dislocations to bypass or cut through reinforcements, and this leads to Orowan strengthening and higher yield strength [86]. Then, during plastic deformation, an increase in the geometrically necessary dislocation density at the interface is induced by graphene, and this results in an additional 54 MPa of flow stress at a 5.8% strain in 0.50 vol % graphene nanosheet/Al [87].

**Figure 4:**
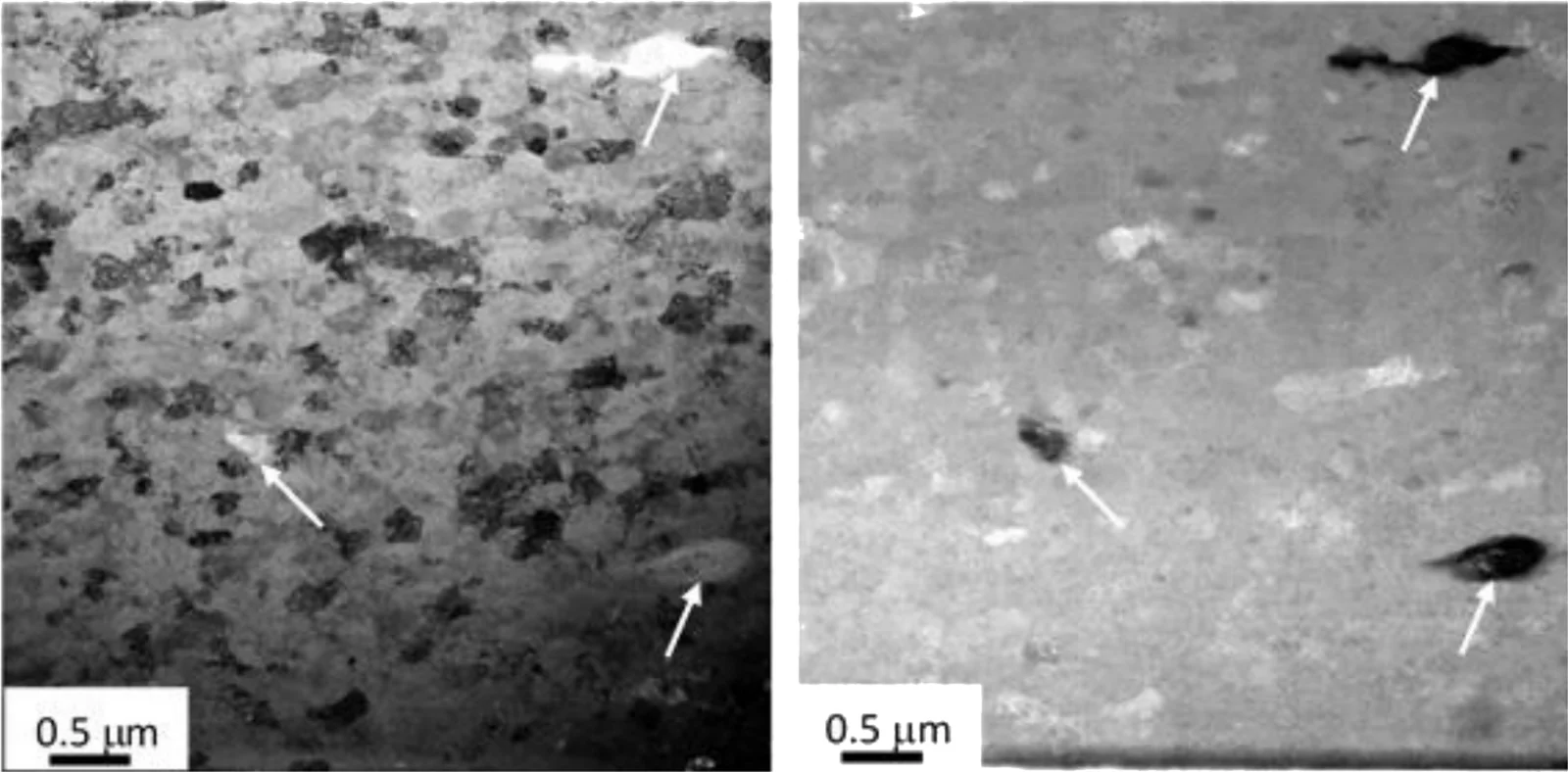
STEM and Z-contrast images showing the microstructures of GNP/Al uniform and linear arrangement processed by HPT at 473 K for *N* = 20 rotations (Reproduced from [90] (© 2018 Y. Huang, et al., published by Elsevier Ltd on behalf of Acta Materialia Inc., distributed under the terms of the Creative Commons Attribution-NonCommercial-NoDerivatives 4.0 International License, http://creativecommons.org/licenses/by-nc-nd/4.0/. This content is not subject to CC BY 4.0).

Electrical and thermal conductivities of graphene/aluminum composites are critically governed by graphene dispersion homogeneity and interfacial properties [34,45,88–90]. According to calculations, the electrical conductivity of Al composites generally increases with graphene adding, and this is attributed to percolating conductive network formation [34]. However, the measured interfacial resistivity is around 10^−8^ Ω·m, and this substantially suppresses theoretical performance gains [34]. After vacuum hot-press sintering of a 0.5 wt % graphene/aluminum composite, graphene was uniformly dispersed at the grain boundaries in the Al matrix. The resulting composite had thermal and electrical conductivities higher by 7.1% and 4.0%, respectively, compared to pure Al [88]. A conductivity test on multilayer graphene/aluminum obtained through high-pressure torsion (HPT) was conducted by Huang and coworkers [90]. The results showed that when 5% graphene is used, the conductivity is significantly increased to 64.9–66.7% IACS, and this surpasses the theoretical conductivity of pure high-purity aluminum. This is attributed to the uniform and linear arrangement of graphene (Figure 4).

Regarding thermal conduction, the interfacial phonon transport mechanism serves as the core factor determining the overall performance of such composites [91–94]. It has been demonstrated that despite the ultrahigh intrinsic thermal conductivity of graphene, composites fabricated by incorporating graphene into matrices of metals often fail to achieve the expected improvement in thermal conductivity, and even a reduction in this property is exhibited. The fundamental bottleneck underlying this counterintuitive phenomenon is the high interfacial thermal resistance (ITR). ITR mainly originates from two effects, namely phonon spectrum mismatch and phonon localization. The former refers to the mismatch in phonon vibration frequencies between metals and graphene, while the latter describes the phenomenon where phonons are confined near the interface. Interfacial bonding characteristics are found to exert a decisive influence on the ITR. In addition, the density and phonon spectrum characteristics of the metal matrix, graphene strain induced by lattice mismatch, and various defects in the interfacial region are found to exert synergistic regulatory effects on phonon transport efficiency. Accordingly, strengthening interfacial phonon coupling is recognized as the key pathway to improve the thermal conductivity of such composites. This conclusion not only provides theoretical guidance for design strategies of high-performance thermal management materials by interfacial alloying, but also lays a theoretical foundation for retarding thermal conduction via structural regulation including constructing disordered interfaces, so that novel thermal insulation materials and thermoelectric materials can be developed.

Employing density functional theory (DFT) and the non-equilibrium Green’s function formalism, phonon transport characteristics and interfacial heat transfer efficiency at metal-encapsulated graphene heterostructures were examined by Tao et al. [95–97] and Chen and coworkers [98–99]. The systems comprised monolayer graphene, double-sided hydrogenated graphene, and single-sided hydrogenated graphene sandwiched between copper and nickel substrates. Their computational results reveal a critical dependency of thermal conductance across metal/Gr/metal interfaces on the nature of graphene–metal bonding. Specifically, chemical bonding (chemisorption) is found to significantly boost interfacial thermal conductance, whereas physical adsorption mechanisms markedly suppress it. To experimentally validate this phenomenon, a novel methodology for enhancing thermal transport at metal–graphite junctions was developed by them. After focused ion beam (FIB) milling pretreatment of graphite substrates, the interfacial thermal conductance between aluminum and graphite was fivefold enhanced [97]. This substantial improvement correlates with the reduction in Fermi level energy observed in milled graphite relative to untreated specimens. The modified electronic structure facilitates stronger interfacial coupling, and phonon-mediated heat transfer across the hybrid interface is thereby optimized. This provides a new feasible approach for further leveraging the conductive and thermal properties of graphene and developing high-performance graphene/aluminum alloys.

### 5 Conclusion, future applications and challenges

Graphene/aluminum composites exhibit exceptional mechanical, thermal, and electrical properties, and they are positioned as transformative materials for high-performance industrial applications. How to produce materials with high performance at a low cost is regarded as the most significant issue from the perspective of industrialization. The industrialization of high-performance Gr/Al composites is currently hindered by the C–Al interface, which is inherently unstable and difficult to regulate. Excessive detrimental interfacial reactions severely impair the strength of the composites; also, they act as a critical barrier to phonon transport. The energy interfacial resistance is pushed to an unacceptably high level. The connection between graphene and aluminum is usually only maintained by weak van der Waals forces, and such adsorption force is insufficient to realize effective load transfer or energy conduction. This is the root of the issue of interfacial bonding.

The most feasible solution is low-cost surface modification of graphene. Instead of directly mixing pristine graphene with aluminum, the graphene surface is coated or modified with a thin and uniform transition layer. This coating must protect the carbon material from erosion by aluminum melt and inhibit the formation of Al_4_C_3_. Besides, it must convert the interface from a region dominated by weak physical adsorption to one with strong chemical bonding. Elements including Cu, Ti, or Ni, deposited on the graphene surface via scalable chemical or electrochemical routes, can form stable thin-layer carbides or intermetallic compounds that act as robust bridges between the two components. This engineered layer can significantly improve phonon coupling and reduce interfacial resistance of energy transfer.

Suitable methods need to be developed to cut the cost of composite processing while performance is maintained. If the carefully constructed interface is damaged during the subsequent consolidation and forming process, even the most sophisticated surface modification will be rendered futile. Conventional powder metallurgy routes involve long-term high-temperature sintering, and this may lead to decomposition or coarsening of the protective coating. Casting is low in cost, but it faces inherent challenges in wetting, dispersion, and uniformity control. Processing innovations that operate at lower temperatures and shorter holding times are required by the industry, such as combining advanced solid-phase processes like SPS with low-cost solutions like stir casting. Because of the requirements regarding solid-state batteries and the thermal management of artificial intelligence chips, the capacity of SPS and other isopressing equipment has been significantly enhanced. These routes can complete composite consolidation rapidly, the fragile interfacial structure is preserved, and uniform dispersion rather than agglomeration of the modified graphene is ensured.

Future research efforts should be prioritized as follows: (1) low-cost protective coatings on graphene to enhance interfacial adhesion through covalent bonding mechanisms, alloy coating, vacancy engineering, hydrogenation, and FIB treatments and (2) rapid low-temperature consolidation methods such as rapid spark plasma sintering combined with casting.

## Data Availability

All data that supports the findings of this study is available in the published article and/or the supporting information of this article.
